# Semi-supervised data-integrated feature importance enhances performance and interpretability of biological classification tasks

**DOI:** 10.1093/bioinformatics/btaf190

**Published:** 2025-07-15

**Authors:** Jun W Kim, Russ B Altman

**Affiliations:** Department of Biomedical Data Science, Stanford University, Stanford, CA 94305, United States; Department of Biomedical Data Science, Stanford University, Stanford, CA 94305, United States; Departments of Bioengineering, Genetics, and Medicine, Stanford University, Stanford, CA 94305, United States

## Abstract

**Motivation:**

Accurate model performance on training data does not ensure alignment between the model’s feature weighting patterns and human knowledge, which can limit the model’s relevance and applicability. We propose Semi-Supervised Data-Integrated Feature Importance (DIFI), a method that numerically integrates *a priori* knowledge, represented as a sparse knowledge map, into the model’s feature weighting. By incorporating the similarity between the knowledge map and the feature map into a loss function, DIFI causes the model’s feature weighting to correlate with the knowledge.

**Results:**

We show that DIFI can improve the performance of neural networks using two biological tasks. In the first task, cancer type prediction from gene expression profiles was guided by identities of cancer type-specific biomarkers. In the second task, enzyme/non-enzyme classification from protein sequences was guided by the locations of the catalytic residues. In both tasks, DIFI leads to improved performance and feature weighting that is interpretable. DIFI is a novel method for injecting knowledge to achieve model alignment and interpretability.

**Availability and implementation:**

Code and models for our experiments are available at https://github.com/junwkim1/DIFI

## 1 Introduction

Convolutional neural networks (CNNs) learn to prioritize important features for various tasks through feature weighting. Beyond helping to improve model performance, interpretability methods can reveal novel insights into biological tasks. DeepFRI, which combines graphical and sequence-based features to predict protein function, has shown that class activation mapping allows function predictions with residue-level annotations (Gligorijević *et al.* 2021). Other CNN models, such as ProteinInfer have used activation mapping to assess the significance of sequence regions and individual residues for specific protein functions (Sanderson *et al.* 2023). In genetics, saliency and activation maps have been used to investigate genes that differentiate cell types and DNA sequences with functional motifs (Shrikumar *et al.* 2019, Mostavi *et al.* 2020). Activation maps have also been used for clinical images to identify important morphological features (Li *et al.* 2019).

Feature weighting varies significantly depending on the model and input data. Studies have reported feature maps that fail to provide explanations when the feature weighting do not align with human perception, and this raises the need for methods to quality control (Jaworek-Korjakowska *et al.* 2021, Huynh *et al.* 2022). Incorporating domain-specific prior knowledge (knowledge injection) into feature weighting improves interpretability by aligning with real-world constraints. For instance, tracking a physician’s attention throughout a clinical image and explicitly incorporating the attention regions into a CNN improves image classification accuracies (Li *et al.* 2019, Mitsuhara *et al.* 2019, Tsuji *et al.* 2023). Such approach has been shown to be effective in other fields such as visual quality assessment and synthetic image detection (Xu et al. 2017, Boyd *et al.* 2023).

For applications in biological research, a key challenge in knowledge injection using constraints on the model’s feature weighting is that there is often an incomplete knowledge and high noise due to the complexity of the system. To incorporate domain knowledge into model feature weighting for biological tasks, we propose Semi-Supervised Data-Integrated Feature Importance (DIFI). DIFI is a novel knowledge injection method that guides the development of feature weighting using task-correlated data, which we refer to as a knowledge map. A knowledge map contains values over input dimensions that indicate the relative importance for the model’s task. These values can be derived from the input data based on a known mechanism or obtained from independent studies. The model is then constrained to develop feature weights correlated with the knowledge map by incorporating the similarity between the knowledge map and the feature map into a loss function. Semi-supervised learning (SSL) of feature weights allows the knowledge map to be sparse and removes the requirement for it to be complete. We hypothesize that as the model learns weights for all features, it compensates for the information missing in the sparse knowledge map through self-learning. Therefore, DIFI enables a balance between exploration and exploitation. To distinguish the values of the learned feature maps from those of the knowledge map, we refer to them as feature weights and knowledge scores, respectively. For clarity, we refer to DIFI involving saliency maps and activation maps as gradient-based DIFI and activation-based DIFI, respectively.

Attention transfer is a method of knowledge distillation, in which a student model’s feature map is constrained by that of a teacher model (Zagoruyko and Komodakis 2017). To simulate sparse knowledge map, we transfer sparse feature map and show that only a small number of highly weighted features is sufficient to improve the performance of the student model. Next, we use DIFI for two biologically relevant tasks: (i) cancer classification with gene expression profiles using gradient-based DIFI and (ii) protein function prediction with protein sequences using activation-based DIFI.

Molecular classification of cancer is fundamental to accurate diagnosis and therapeutic decisions. The differential gene expression (DGE) analysis is a core transcriptional analysis method that measures changes in gene activities and encapsulates extensive biological knowledge of cancer (Mostavi *et al.* 2020). To apply DIFI to the transcriptomics-based cancer classification task, we generate a knowledge map for each training sample by assigning high knowledge scores that correlate with expression levels of cancer type-specific biomarkers. The non-biomarker genes were not assigned scores, allowing the model to implicitly learn feature weights. The models with DIFI show improvements in accuracy and generate feature weighting that correlate with the biomarkers.

To evaluate DIFI on protein classification, we apply DIFI on a model adopted from ResNet18 for protein sequence-based enzyme or non-enzyme prediction. High knowledge scores are assigned to residue positions that are known to be functionally important (catalytic). Consistent with the cancer classification task, DIFI improves protein classification and residue-level interpretability. We find that the model outputs are highly sensitive to mutations of the predicted functional residues, an observation generally reported in experimental studies. Thus, DIFI is a novel approach to align neural networks with *a priori* knowledge represented as task-associated data, improving performance, interpretability, and relevance.

## 2 Materials and methods

### 2.1 DIFI learning objective

Data sparsity is often introduced to biological datasets due to noise and complexity of the system. SSL tackles incomplete information and posits that, even unlabeled samples can help predictions (Chapelle *et al.* 2009). If we use X, Y, and $X^{u}$ to denote an input sample, a predicted label, and an unlabeled sample, respectively, SSL aims to improve P(Y|X) with $X^{u}$ the cluster assumption, which posits that unlabeled samples forming the same cluster with a set of labeled samples should share the sample label. Analogously, for a sample *X*, if we use $W_{X}$, $F_{X}$, and $F_{X}^{u}$ to denote the learned feature weighting, a feature, and a feature without an assigned weight, respectively, in DIFI we aim to improve $P(W_{X}|F_{X})$ with $F_{X}^{u}$ by applying the cluster assumption. Specifically, we assume that features in the same cluster of $P(F_{X})$ should have the same $W_{X}$.

**Figure 1.**
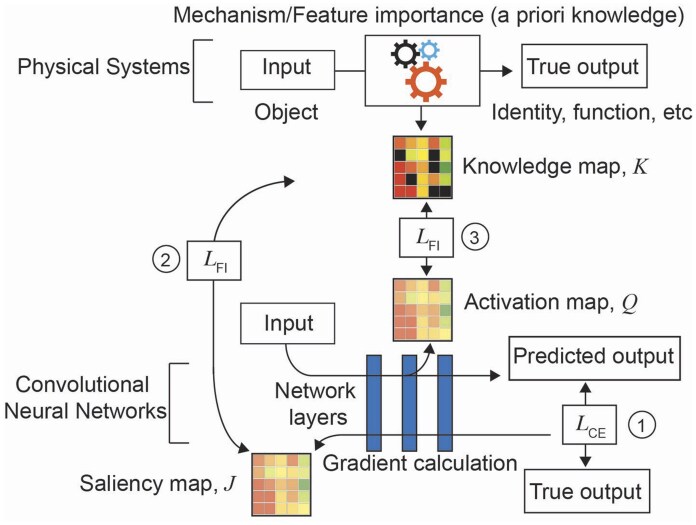
Schematic representation of knowledge injection with DIFI. In physical systems, entities and their associated function are identified based on known features. For DIFI, these features are stored as a knowledge map and incorporated into a CNN through its feature maps, enabling the model to learn feature weighting that align with *a priori* knowledge. In biological systems, knowledge maps can be sparse (black squares: missing data). We hypothesize that DIFI leverages existing knowledge while filling in the gaps as the model trains to perform the primary prediction task. 1: Standard cross entropy loss (CE). 2: The knowledge map and the saliency map similarity (FI, feature importance). 3: The knowledge map and the activation map similarity.

To describe the DIFI method (Fig. 1) we use the two types of features maps used in attention transfer: saliency map (gradient-based) and activation map (activation-based) and follow the convention described in (Zagoruyko and Komodakis 2017). In a saliency map, the saliency value of each feature is a gradient w.r.t. the input. Let W and x denote model weights and input data, respectively, and let K denote *a priori* knowledge in the form of numerical data. The strategy for preparing K should be determined based on the task, as we describe for each case. In this study, we use feature importance values derived from the input data or an independent database. Since we want the model’s feature weighting to develop a pattern similar to that of the a prior knowledge, we minimize a vector distance between them. We define the objective function for gradient-based DIFI, $L_{\text{GB}}$, as:

$$L_{\text{GB}}=L_{\text{CE}}(W,x)+\alpha ∥J_{M}-K_{M}{∥}_{2},\;\text{where}$$

 

$$K_{Mi}=\theta_{i}K_{i}$$

 

$$J_{Mi}=\theta_{i}J_{i},J_{i}=\frac{\partial }{\partial x}L_{\text{CE}}(W,x_{i})$$

$\theta_{i}$ is 0 if $K_{i}$ does not satisfy the threshold percentile and/or significance, masking these regions. α is the weight of similarity between the knowledge map and the feature map.

In an activation map, consider an activation tensor A. An activation-based mapping function F takes A and outputs a spatial activation map with the same dimension as the input. Then we can define the objective function of activation-based DIFI, $L_{\text{AB}}$, as:

$$L_{\text{AB}}=L_{\text{CE}}(W,x)+\alpha ∥Q_{M}^{j}-K_{M}{∥}_{2},\;\text{where}$$

 

$$Q_{M}^{j}{}_{i}=\theta_{i}Q_{i}^{j},Q_{i}^{j}=F{\left(A^{j}\right)}_{i}$$

where $Q_{i}^{j}$ is the i-th element of the j-th activation map. For the distance metric, we use $l_{2}$ distance and cosine similarity, but other distance metric can be employed as well. We keep the standard cross-entropy loss, $L_{\text{CE}}(W,x)$ for the performance of the model. For convenience, we refer to both saliency map values and activation map values as feature weights.

### 2.2 Sparse attention transfer objective

To evaluate the feasibility of incorporating *a priori* knowledge in semi-supervised manner, we first conducted experiments using a synthetic knowledge data based on attention transfer. The attention transfer used for knowledge distillation (Zagoruyko and Komodakis 2017) involves supervised learning of feature weighting using a complete feature map. The integration of biological data into a saliency map is expected to involve sparse data. Although sparse features can be problematic, a small set of well-selected features can improve performance (Lemhadri *et al.* 2021). To simulate DIFI with sparse *a priori* knowledge we only use the features with the top saliency values for each sample (Supplementary Fig. S1). Let S, T and $W_{S}$, $W_{T}$ denote student, teacher and their weights correspondingly, as used in gradient-based attention transfer (Zagoruyko and Komodakis 2017). We define the objective function for the simulation, $L_{\text{Sim}}$.

$$L_{\text{Sim}}=L(W,x)+∥J_{\text{SM}}-J_{\text{TM}}{∥}_{2},\;\text{where}$$

 

$$J_{\mathrm{SMi}}=\theta_{i}(J_{Si}),J_{\mathrm{TMi}}=\theta_{i}(J_{Ti})$$

 

$$J_{Si}=\frac{\partial }{\partial x}L(W_{S},x_{i}),J_{Ti}=\frac{\partial }{\partial x}L(W_{T},x_{i})$$

$\theta_{i}$ is 0 if $S_{\text{Ti}}$ does not satisfy the threshold percentile, masking these features for sparsity. We refer to attention transfer involving sparse feature map as sparse attention transfer.

### 2.3 Image model architecture and learning

We train a shallow CNN and a network-in-network (NIN) using a small binary image classification dataset and CIFAR-10 using Keras to perform binary and multiclass classification task (Supplementary Fig. S1). For simplicity, the first proof-of-concept (POC) study involves a model with a small architecture consisting of two convolutional layers, 2D max pooling, followed by a fully connected layer. The teacher (tCNN, 985 parameters) and student (sCNN, 521 parameters) models are trained for 100 epochs and 150 epochs, respectively, using a batch size of 64. For saliency map generation, each sample is fed into the trained teacher model and feature weight for each input pixel is generated by computing the gradient of the loss w.r.t. the input pixel. As the student model is trained, only the pixels within the selected percentile of the feature weight are used to transfer by minimizing the loss function using l2 distance metric. For the CIFAR-10 experiment with NIN model, 3-layer teacher model (tNIN, 545,024 parameters) and 3-layer student NIN model (sNIN, 51,702 parameters) are trained for 30 epochs and 50 epochs, respectively, using a batch size of 64.

### 2.4 Cancer model architecture and learning

To simulate DIFI for cancer cell classification using gene expression data, we implement 1DCNN from Mostavi *et al.* (2020) using Keras. 1DCNN is a shallow CNN model that takes the gene expression profile as a vector. It applies one-dimensional kernels to the input, followed by a 2D max pooling layer and a fully connected layer, and a prediction layer. Such a shallow architecture is preferred over deep neural networks when dealing with small sample sizes to prevent overfitting (Mostavi *et al.* 2020). Additionally, for gradient-based feature map generation, a shallow architecture requires fewer computations for generating second-order mixed partial derivatives during the second propagation step, which updates the network weights based on $∥J_{SM}-J_{TM}{∥}_{2}$. We simulate DIFI using sparse attention transfer from the teacher model (t1DCNN, 4 368 929 parameters) to the student model (s1DCNN, 99 579 parameters). The teacher and student models were trained for 50 epochs and 100 epochs, respectively, using a batch size of 128.

**Figure 2.**
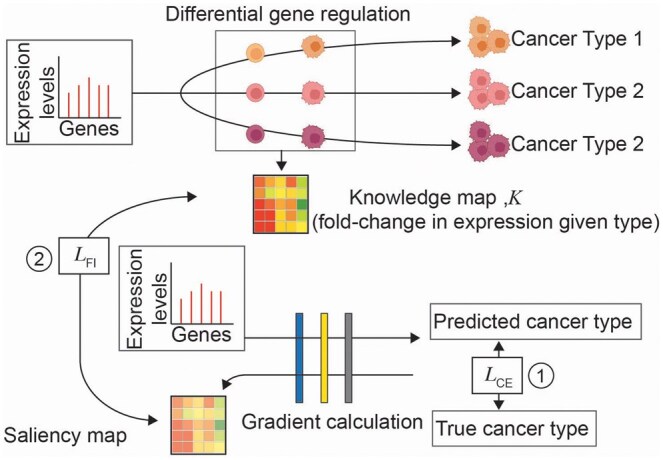
Schematic representation of DIFI for cancer classification. Each patient/tumor/cancer cell has differentially regulated genes that lead to a specific cancer type. Such information can be represented as a knowledge map, and for cancer classification overexpressed genes are used. 1DCNN architecture has one-dimensional kernels to the input, followed by a 2D max pooling layer and a fully connected layer. 1: Standard cross entropy loss. 2: l2 distance.

For evaluating DIFI, biomarker genes for each sample are determined from the genes specifically overexpressed with statistical significance (*P*-value < .05) in the cancer type of the sample. The overexpression levels of these markers are standardized and used as knowledge scores for each cancer type. During the training, each training sample is matched with a knowledge map based on its cancer type (Fig. 2). We use grid search to identify the optimal parameters. The search space for the number of biomarker genes is {20, 10, 5, 2}. The search space for α is 0.3, 1, 3, 10. The input data are divided into training (90%) and testing (10%). We extract 20% of the training set to form a validation set, which results into 72/18/10 split of the groups for training/testing/validation.

### 2.5 Protein model architecture and learning

We train ResNet18-based model using PyTorch to classify protein sequences as enzymes or non-enzymes due to its clear advantages. ResNet18 is organized into four groups of convolutional layers, with each group containing two residual blocks. This structure allows us to generate activation maps at various layers and examine feature weighting at different depths (Fig. 4). Another key architectural advantage of ResNet18 for DIFI is skip connections. Within each group, skip connections bypass two convolutional layers. This ensures that highly selective feature weighting at a particular layer does not eliminate contributions from less-weighted features, because the bypassed information is carried over from the previous layers through the skip connections. The inputs are protein sequences with each residue encoded using one-hot encoding. To reduce training time, we limit the maximum protein length to 300. To constrain feature weighting, Activation Map 3 is generated from the output of the Group 3 layers (Fig. 4). To view whether DIFI is preserved at a deeper layer, Activation Map 4 is generated from the output of the Group 4 layers. The activation maps are generated only using average pooling (a non-trainable operation) to propagate SSL throughout the model’s feature weighting. The input data are divided into training (90%) and testing (10%). We extract 20% of the training set to form a validation set, which results into 72/18/10 split of the groups for training/testing/validation. Knowledge maps are generated using the catalytic site dataset.

**Figure 3.**
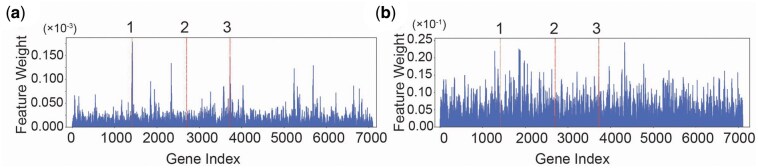
Visualizing feature weighting of s1DCNN_3_. (a) Saliency map of a TCGA-BRCA sample using s1DCNN_3_. (b) Same as in (a) but using s1DCNN without DIFI. Red dotted lines denote indices of the biomarker genes for TCGA-BRCA. 1: SCGB2A2; 2: LMX1B; 3: PIP.

For each enzyme, the known catalytic positions (labeled knowledge data) are used to generate the knowledge map. These positions are assigned a knowledge score of 1. To favor relatively higher weights for the known positions, a number of residues are randomly selected from the remaining positions and assigned a knowledge score of 0.01. Then, we compute the pairwise cosine similarity between the knowledge map and Activation Map 3, along with a standard cross-entropy loss (Fig. 3). For each epoch, the low knowledge score is reassigned randomly among the unknown positions to allow the screening of residues (unlabeled knowledge data) for higher weights. We use grid search to identify the optimal parameters. The search space for the number of random residues is 3, 30, 60, 100 corresponding to 1%, 10%, 20%, and 33% of maximum length. The search space for α is 0.3, 1, 3, 10. To train a control model (ResNet18-Ran_3_) with non-functional knowledge maps, DIFI is applied with a knowledge map that has randomly assigned feature weight of 1. The number of residues assigned for each sequence was the same as that of the known catalytic positions.

Perturbation experiments are conducted using alanine substitutions. Within the test set, a separate set of sequences that are correctly identified as enzymes are prepared. For each protein sequence in this set of true positives, residues are selected based on the feature weights generated by the trained models and replaced with alanine. Percent conversion is calculated as the percentage of true positive sequences that are predicted as non-enzymes following alanine substitutions.

### 2.6 Knowledge map incorporation

To incorporate a knowledge map for cancer and protein classifications, assume that we have an input vector $x=[x_{1},\ldots ,x_{d}]\in R^{d}$, where d is the number of features, and $M=\{x_{i}|x_{i}\;\text{satisfies}\;\text{the}\;\text{filtering}\;\text{condition}\}$. The feature filter, θ, can be expressed as a vector where

$$\theta_{i}=\{\begin{array}{cc} 1, & \text{if}\;x_{i}\in M,\\ 0, & \text{otherwise}. \end{array}$$

The feature maps and the knowledge map are filtered as

$$\begin{array}{c} J_{M}=J⊙\theta ,\\ Q_{M}=Q⊙\theta ,\\ K_{M}=K⊙\theta , \end{array}$$

where ⊙ represents the Hadamard product. Here, J and Q are gradient-based and activation-based feature maps, respectively, as previously defined. $K=[k_{1},\ldots ,k_{d}]\in R^{d}$ is numerical data representing *a priori* knowledge (K) and referred to as the knowledge map. Thus, there are two key considerations when preparing a knowledge map. The first is the importance of the knowledge K for the task, necessitating evaluation by a domain expert. The second is the quality of the knowledge, which can be assessed and filtered by θ using inference.

For the cancer classification task, DGE is a well-established mechanism driving cancer phenotypes, and the fold change values were used for K. The set M was selected based on whether the *P*-value of the fold change was less than 0.05. For the protein classification task, annotations from UniProt, which are generally accepted as predictions of catalytic residues, were used for M. For K, values of 1 and 0 were assigned to catalytic and unlabeled sites, respectively.

### 2.7 Dataset details

A small dataset from Stanford CS230: Deep Learning is used to simulate DIFI for binary classification tasks. The dataset comprises 259 images, each sized 64 × 64 pixels images labeled as 1 (cat) or 0 (non-cat) (Ng *et al.*  https://www.coursera.org/specializations/deep-learning].

CIFAR dataset, containing 60 000 images of size 32 × 32 pixels, is used to simulate DIFI for multi-class classification tasks (Krizhevsky 2009).

Gene expression profiles from the Cancer Genome Atlas (TCGA) are used to evaluate DIFI in the cancer classification task (Mostavi *et al.* 2020). 10 340 cancer samples are used to train models for predicting of 33 cancer types and conducting DGE analysis. The preprocessed data are obtained from the study titled *Convolutional neural network models for cancer type prediction based on gene expression* (Mostavi *et al.* 2020). Briefly, pan-cancer RNA-Seq data from TCGA was downloaded by Mostavi *et al.* through an R/Bioconductor package TCGAbiolinks and represented as ${\;\text{log}\;}_{2}$(FPKM + 1), where FPKM is the number of fragments per kilobase per million mapped reads, and genes with low information burden were removed.

The catalytic site dataset is obtained from the study, *Explainable protein function annotation using local structure embeddings* (Derry and Altman 2023). Briefly, we use the “gold-standard” evaluation dataset, which was prepared by identifying enzymes in SwissProt using sequence homologs in the Enzyme Catalytic Site Atlas (CSA) and further filtering to keep only those with perfectly matching EC number for the reference in CSA. Non-enzyme dataset is obtained from the study titled *Sequence-based enzyme EC number prediction by Deep Learning (DEEPre) [Li et al. 2018]. Briefly, 22,168 non-enzyme sequences were randomly selected from the SwissProt database. Those annotated with “fragment” or with <50 residues were removed and rest of the sequences were sifted with 40% similarity threshold.* To prevent bias, the number of enzyme and non-enzyme sequences was matched with random selection before use.

## 3 Results

### 3.1 Sparse attention transfer

To test our hypothesis that SSL with sparse dataset is sufficient to guide feature weighting, we conduct two POC experiments using image classification tasks. To simulate a sparse dataset, we use attention transfer with sparse saliency maps, where only the pixels corresponding to a selected percentile of the saliency values for each input are transferred from the teacher model to the student model (Supplementary Fig. S1). In the first experiment, the teacher model (tCNN, 985 parameters) is trained for a binary classification task for images labeled as cat or non-cat. For each sample, pixels corresponding to a selected percentile of the saliency values are transferred from tCNN to the student model (sCNN, 521 parameters). The results can be found in Supplementary Table S1. Transferring the entire saliency map from tCNN to sCNN decreases the performance of sCNN, rather than enhancing. In contrast, selectively transferring the top 10%, 5%, and 2% values leads to sCNN surpassing the performance of the base model, which was not trained with attention transfer.

To evaluate SSL with a larger and deeper model for multiclass image classification task, we use 3-layer network-in-network (NIN) models (Lin *et al.* 2014) on CIFAR-10 dataset. As above, pixels corresponding to saliency values of top percentiles ranging from 50 to 2 are transferred from the teacher model (tNIN, 545 024 parameters) to the student model (sNIN, 51 702 parameters). As shown in Table 1, transferring the top 10%, 5%, and 2% percentile values improves the performance compared to the base model, consistent with our observation from the binary classification task.

**Table 1.** Performance of NIN with gradient-based sparse attention transfer on CIFAR-10.^a^

Model	Student	Sim_100_	Sim_50_	Sim_10_	Sim_5_	Sim_2_
Error	28.74	30.55	28.79	24.33	20.25	22.81

a Error is computed as median of 3 runs. The subscripts denote the percentages of the total number of pixels used for attention transfer.

### 3.2 Cancer classification

Various next-generation sequencing (NGS) platforms are available for cancer diagnosis, prognosis, and therapeutics. Deep learning models utilizing NGS data have been developed to predict cancer types and patient survival, and those specifically developed for improved interpretability allows additional insights on cellular traits and disease (van Hilten *et al.* 2021). To the best of our knowledge, neither attention transfer nor knowledge injection has been used with biological or NGS data.

To further evaluate the feasibility of DIFI for NGS data, we simulate DIFI using sparse attention transfer. We use 1DCNN architecture from Mostavi *et al.* (2020). The teacher model (t1DCNN, 4 368 929 parameters) was trained to classify 33 TCGA cancer types. Then a selected percentile of the saliency values were transferred from t1DCNN to the student model (s1DCNN, 99 579 parameters). Consistent with the image classification simulations, selective attention transfers improved cancer classification accuracy, with the highest improvement observed using the top 5 saliency values (Supplementary Table S2).

Having confirmed effectiveness of sparse attention transfer, we evaluate DIFI. Biomarker genes for each of the cancer types are determined from overexpressed genes identified from DGE analysis. We represent the normalized expression levels of the selected number of the most expressed genes as a knowledge map for each cancer type and integrate into the model’s feature weighting (Fig. 2). The results can be found in Table 2. Selectively guiding only the top 50 to 2 most overexpressed gene for each of the cancer types substantially improves performance, with guiding 3 genes (s1DCNN_3_) leading to an error lower than that of the base model by nearly 22%.

**Table 2.** Performance of 1DCNN with gradient-based DIFI on cancer classification.^a^

Model	s1DCNN	s1DCNN_20_	s1DCNN_10_	s1DCNN_3_	s1DCNN_2_
Error	33.06	16.4	15.75	10.84	11.48

a Error is computed as median of 3 runs. The subscripts denote the number of genes used for DIFI.

We visualize feature weighting of s1DCNN_3_ through the feature map of the trained model. Feature map of a representative sample from a BRCA (breast cancer) patient assigns the 2 out of 3 biomarker genes, SCGB2A2 and PIP, with the top 10 feature weights (Fig. 3a). Indeed, SCGB2A2 and PIP are known biomarkers for breast cancer (Iman *et al.* 2020, Sauer *et al.* 2023). In contrast, the base model did not exhibit selectively high feature weighting of the biomarker genes (Fig. 3b).

### 3.3 Protein classification

**Figure 4.**
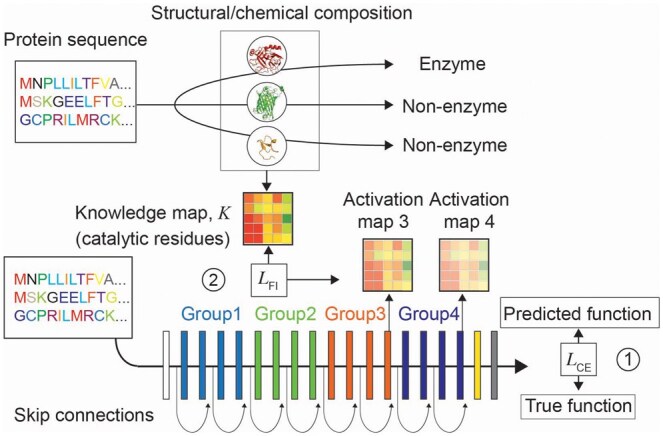
Schematic representation of DIFI for protein classification. Each protein sequence has a unique structural and chemical information that leads to the protein function. Such information can be represented as a knowledge map, and for enzyme/non-enzyme classification positions of catalytic residues are used. ResNet18 architecture has four groups of convolutional layers. Each group has two residual blocks. Skip connection bypasses two convolutional layers. Activation map 3 is derived from the output from group 3 and constrained by the knowledge map. 1: standard cross entropy loss. 2: cosine similarity.

Experimental validation of protein function requires time-consuming synthesis and purification steps that need to be optimized for each protein. Computational prediction of protein classification and precise location of key residues can facilitate efficient experimental design, protein engineering, and drug development. Given a model that predicts function from protein sequences, feature maps have been shown to correlate with known functional domains, suggesting that increased feature weighting to functionally important residues contributes to prediction performance (Gligorijević *et al.* 2021, Sanderson *et al.* 2023). To evaluate DIFI for protein classification task, we trained ResNet18-based model to predict whether the provided protein sequence is an enzyme or non-enzyme (Fig. 4). The function of a protein depends on one-dimensional as well as higher order structures, and the convolutional architecture of ResNet18 and activation-based DIFI is well suited. The ResNet architecture has skip connections and convolutional layers organized by groups, which allow knowledge map integration at different layers. Additionally, skip connections can help preserve information even from less attended residues. We choose Group 3 for sufficient depth and leave Group 4 to examine the preservation of the introduced knowledge.

To examine the effect of DIFI on protein classification, we represent positions of the catalytic residues as a knowledge map and integrate into the model’s feature weighting (Fig. 4). For each enzyme and each epoch, a low knowledge score is assigned to randomly selected 20% of the total residues that are not labeled as functionally important to maintain relatively higher feature weights in the labeled residues. While this dampens the feature weighting in the randomly selected residues, they are allowed to regain feature weights in epochs where they are not selected if they improve the model performance. Table 3 shows the results from applying DIFI with the test set. Without DIFI, the base ResNet18 (RN) exhibited an error of 12.56%, whereas DIFI-applied model with α (the weight of similarity between the knowledge map and the feature map in the loss function, $L_{\text{AB}}$) of 0.3 (RN0.3), 1 (RN1), 3 (RN3), and 10 (RN10) achieved 11.76%, 9.94%, 8.93%, and 9.85%, respectively, with RN3 showing the highest improvement.

**Table 3.** Performance of ResNet18 with activation-based DIFI on enzyme/non-enzyme classification.^a^

Model	RN	RN0.3	RN1	RN3	RN10	Ran3
Error	12.56	11.76	9.94	8.93	9.85	13.52

a RN, ResNet. The numbers in the model names denote the values of α. Error is computed as median of 3 runs.

**Figure 5.**
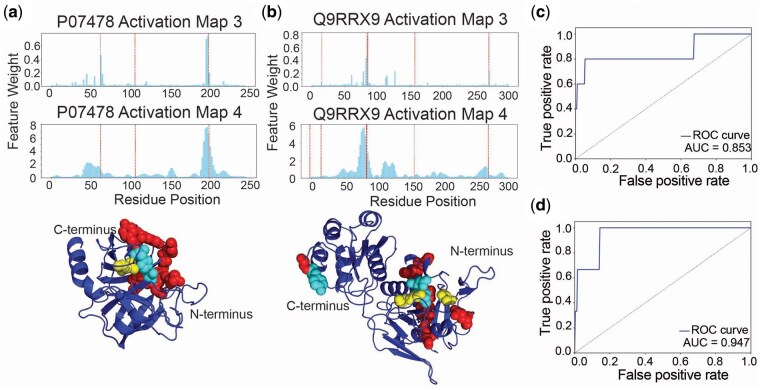
Visualizing feature weighting of RN3. (a) Activation Map 3 and Activation Map 4 of trypsin-2 from RN3 and AlphaFold structure of trypsin-2 (bottom). (b) Activation Map 3 and Activation Map 4 of L-asparaginase from RN3 and AlphaFold structure of L-asparaginase (bottom). (c) ROC curve of trypsin-2 showing the overlap between feature weighting profile from Activation Map 3 and spatial proximity to the catalytic sites. (d) As in (c) but with L-asparaginase. Red dotted lines in (a) and (b) denote the known catalytic residues. In (a) and (b), catalytic residues are shown in yellow. Cyan highlights residues that have among the top 10 highest feature weights and are known catalytic residues. Red indicates other residues with the top 10 highest feature weights.

We visualize feature weighting of RN3 through Activation Map 3 and Activation Map 4 of the two representative proteins from the training set: trypsin-2 (P07478) and L-asparaginase (Q9RRX9). Activation Map 3 of RN3 assigns 2 of the 3 known catalytic residues of trypsin-2 with the top two feature weights (Fig. 5a). Although DIFI is applied in Group 3 using Activation Map3, Activation Map 4 maintains relatively higher feature weights for the catalytic residues, albeit the smoothened distribution, suggesting that the increased feature weighting on these residues are preserved throughout the deeper layers. In the AlphaFold structure of trypsin-2, the residues with 10 highest feature weights are found to overlap or be spatially close to the catalytic residues. Activation Map 3 of RN3 also assigns 2 of the 4 known catalytic residues of L-asparaginase with the top three feature weights (Fig. 5b). Activation Map 4 and AlphaFold structure of L-asparaginase show the similar results to those of trypsin-2.

**Figure 6.**
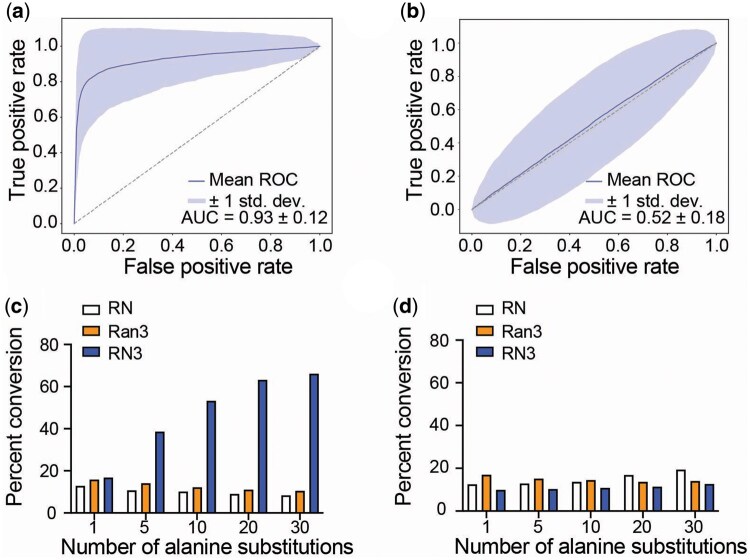
Relevance of high feature weights. (a) Average ROC curve of the test set from RN3 showing the overlap between feature weighting profile from Activation Map 3 and spatial proximity to the catalytic sites. (b) As in (a) but from RN. (c) The percentage of the test set sequences with converted predictions (from enzyme to non-enzyme) when the residues with the top feature weights are substituted with alanine. (d) As in (c) but when the residues with the lowest feature weights are substituted with alanine.

Consistent with these results, when activation site residues are defined as those within 3Å of the known catalytic residues, RN3 showed the ROC-AUC scores of 0.8532 and 0.9474 for trypsin-2 (Fig. 5c) and L-asparaginase (Fig. 5d), respectively. Supplementary Fig. S2a and b shows Activation Map 3’s of representative proteins from the test set. Again, human thymidylate synthase (P04818) and drosophila enolase (P15007) show relatively high feature weights for the known catalytic residues with the ROC-AUC scores of 0.9853 and 0.9932, respectively (Supplementary Fig. S2c). Across the entire test dataset, RN3 achieves average ROC-AUC score of 0.93 while 0.52 is computed for RN (Fig. 6a and b). Without DIFI, Activation Map 3 shows a generally uniform distribution of activation scores throughout the residues, as shown with trypin-2 in Supplementary Fig. S3a.

In contrast to highly reliable *a priori* knowledge, we hypothesize that inaccurate biological knowledge and DIFI can mislead the model’s feature weighting and performance. To test our hypothesis, we examine another ResNet18-based model (Ran3) trained for the enzyme/non-enzyme classification task with the α of 3, this time incorporating false catalytic residues (randomly selected residues). Ran3 does not show an improvement in performance, as expected (Table 3). Interestingly, Activation Map 3 of Ran3 does not exhibit higher feature weighting for either the true catalytic residues or the random residues, suggesting that the model achieves less optimal convergence for the loss function (Supplementary Fig. S3a). Ran3 shows ROC-AUC score of 0.48 for identifying the activation site residues of the test set (Supplementary Fig. S3b).

Leading structure prediction methods such as AlphaFold and ESMFold can accurately predict the overall structures of most proteins, but they are not sufficiently sensitive to accurately predict the changes caused by a small number of missense mutations (Buel and Walters 2022). Sensitivities that mimic those of biological systems can enhance the relevance and capabilities of prediction models. We hypothesize that, with increased feature weighting to active site residues, DIFI leads to a biologically relevant sensitivity to substitutions. Alanine substitution removes side chain interactions, and alanine-scanning is a well-established experimental method to examine the contribution of each residue.

In the test set, we quantify the percentage of sequences with converted predictions-from enzyme to non-enzyme when the residues with the top feature weights are substituted with alanine. In RN3, increasing the number of alanine substitution leads to increased conversion, with five alanine substitutions sufficient to convert ∼40% of the sequences (Fig 6c). In contrast, alanine substitutions for residues with the lowest feature weights did not convert the prediction (Fig. 6d). Without DIFI increasing the number of alanine-substituted residues with high feature weights from RN does not lead to notable changes. Similar results are shown with Ran3, suggesting that the increased sensitivity to the substitutions in the highly weighted residues is specific to RN3.

## 4 Discussion

Knowledge injection via feature weighting is an effective approach to incorporate prior/domain knowledge to improve interpretability and performance (Xu *et al.* 2017, Li *et al.* 2019, Mitsuhara *et al.* 2019, Boyd *et al.* 2023, Tsuji *et al.* 2023). In this work, we investigated guiding feature weighting by incorporating genetic and molecular data. Compared to tasks that involved supervised learning of feature weighting, biological data are generally less complete and noisy. We propose integrating biological data through semi-supervised feature weighting (DIFI), where *a priori* knowledge is represented sparsely as a knowledge map, and the model feature weighting is represented as a feature map.

We examined the effects of DIFI for knowledge injection in three areas: performance, interpretability, and implicit learning. The initial POC experiments involving sparse attention transfer show that a small fraction of the features are sufficient or more effective for knowledge distillation between a teacher and a student model. This observation is critical for biological data, which often require inference, further reducing data points that can be incorporated into a knowledge map. Additionally, biological systems often lead to either a partial understanding or a phenomenon that can be too complicated to be represented as a complete knowledge map. DIFI effectively improves performance of cancer type classification and enzyme/non-enzyme classification tasks using limited knowledge data. The performance of the t1DCNN, ∼40 times larger, and other reported models, can classify cancer more accurately without applying DIFI (Mostavi *et al.* 2020, Gupta *et al.* 2022). However, the improvement of nearly 22% with DIFI over the base model with the equivalent size suggests that DIFI can be an effective approach. The accuracies enzyme/non-enzyme classification using ResNet-based models with DIFI were comparable to or better than previously reported values (Naik *et al.* 2007, Munteanu *et al.* 2008). However, a more controlled study is needed to determine where RN stands among the state-of-the art methods.

Despite the examples of deep learning models that align with human perception or knowledge, interpretability is not guaranteed. For example, feature maps on medical images show that seemingly accurate models can attend to irrelevant features (Jaworek-Korjakowska *et al.* 2021, Huynh *et al.* 2022). With DIFI, s1DCNN effectively learns to increase feature weighting to the overexpressed biomarkers for each cancer type. We show that without DIFI, a enzyme/non-enzyme classifier based on ResNet18 does not increase feature weighting to catalytic residues while DIFI using Activation Map 3 leads to feature maps that correlate with the catalytic residues. The correlated feature weighting is also observed in a deeper layer, as shown by Activation Map 4, suggesting that the feature weighting is preserved throughout the model. A similarly high ROC-AUC score of residue-level interpretability was achieved with DeepFri (Gligorijević *et al.* 2021). Although larger architectures may lead to implicit learning of feature weighting aligned with a prior knowledge, DIFI offers a computationally cost-effective alternative approach.

We expected that using DIFI enable the model to implicitly learn knowledge-aligned weighting also for features not covered by the sparse knowledge map, extending the cluster assumption to feature weighting. Indeed, the activation maps and 3D structures of proteins demonstrate that the model increases feature weights to residues that are both sequentially and spatially closer to the known catalytic residues, even though this information was not provided by the knowledge map. A perturbation experiment using alanine substitution confirms that these residues contribute to the prediction outcome and shows that, even without an alanine-scanning dataset, DIFI leads to sensitivity that could be more biologically relevant.

DIFI can minimize errors by utilizing a small number of carefully selected features, but an important consideration is the impact of inaccurate biological knowledge. To simulate a scenario with completely inaccurate knowledge, we tested the use of random feature importance for the protein classification task. In this case, our model did not demonstrate meaningful learning of feature importance; the activation maps did not correlate with either the catalytic residues or the random residues, suggesting that the model could not converge for the loss function. DIFI with random residues does not completely eliminate accuracy, indicating that the effect of inaccurate knowledge may depend on parameters such as α, integration layer, and the model architecture. For example, the skip connections may exhibit self-correcting behavior, as suggested by the lack of correlation between the randomly assigned residues and Activation Map 3 of Ran3. However, this result does not rule out the risk of the model exhibiting seemingly reasonable performance with false knowledge. Therefore, evaluation of a domain-expert remains critical.

Applications of DIFI to other types of models are evident. Transformer protein language models can also learn to generate attention maps that reflect protein structure, and training the attention heads guided by DIFI (e.g. using experimental structural data) may lead to improved attention mechanisms. We used one-hot encoding to represent genes and protein sequences to highlight the benefits of using DIFI, but embeddings from pretrained models such as ESM offers another approach that can readily benefit from larger models (Lin *et al.* 2023). Generative models are enabling *in silico* protein design with unprecedented accuracy. Similar to classification tasks, feature maps facilitate the interpretation of the generative process (Park *et al.* 2024). Applying DIFI to generative models could provide an exciting new approach to enhance accuracy, biological relevance, and interpretability.

## 5 Conclusion

As we explore using deep learning for natural sciences, integration of prior knowledge becomes more important. Knowledge-integrated frameworks, such as physics-informed neural networks, have shown improvements in various tasks (Raissi *et al.* 2019). DIFI is a novel framework that is compatible with different feature maps and data types, and architecturally less restricted, and facilitates the development of biologically relevant models with efficiency and capabilities.

## Supplementary Material

btaf190_Supplementary_Data

## Data Availability

The data and code underlying this article are available in GitHub at https://github.com/junwkim1/DIFI
