# Supplementary figures and images for: Semi-supervised data-integrated feature importance enhances performance and interpretability of biological classification tasks
Source: Bioinformatics. 2025 Jul 15;41(Suppl 1):i373–81. doi: 10.1093/bioinformatics/btaf190 (PMC12261460; doi:10.1093/bioinformatics/btaf190)

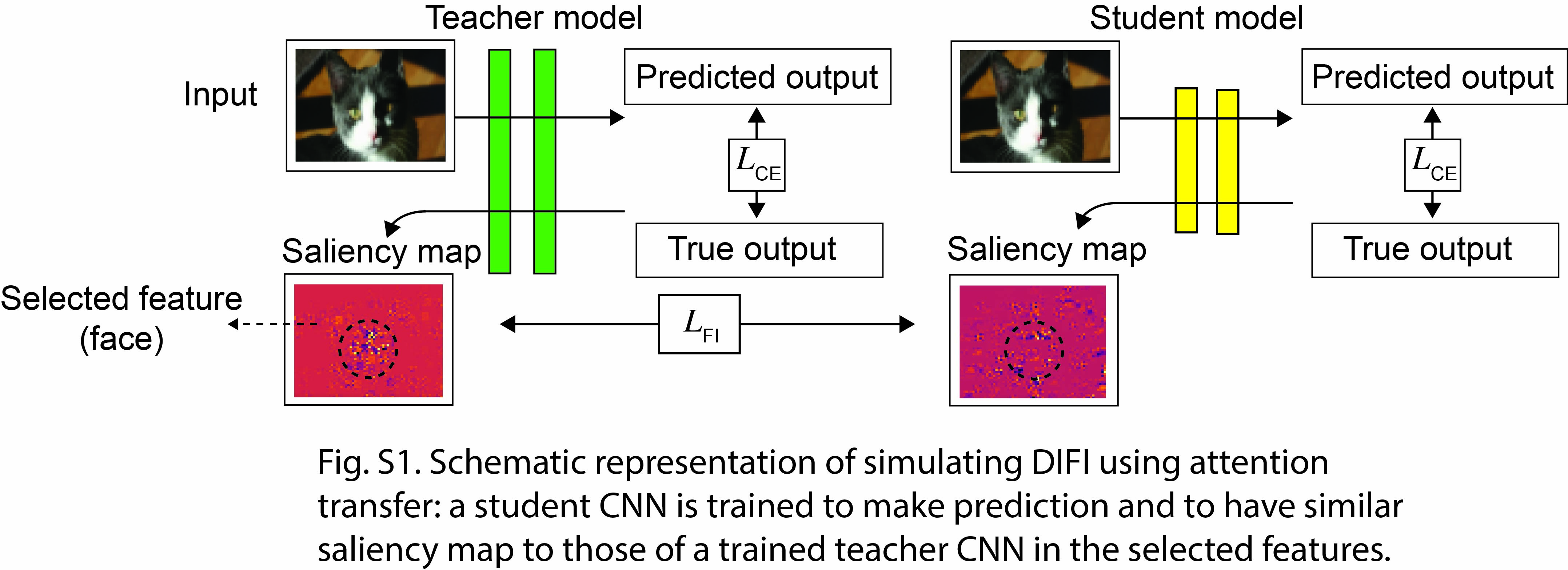

Supplement: btaf190_Supplementary_Data [file btaf190_supplementary_data.zip › btaf190_Supplementary_Data/Kim.50.sup.1.jpg]

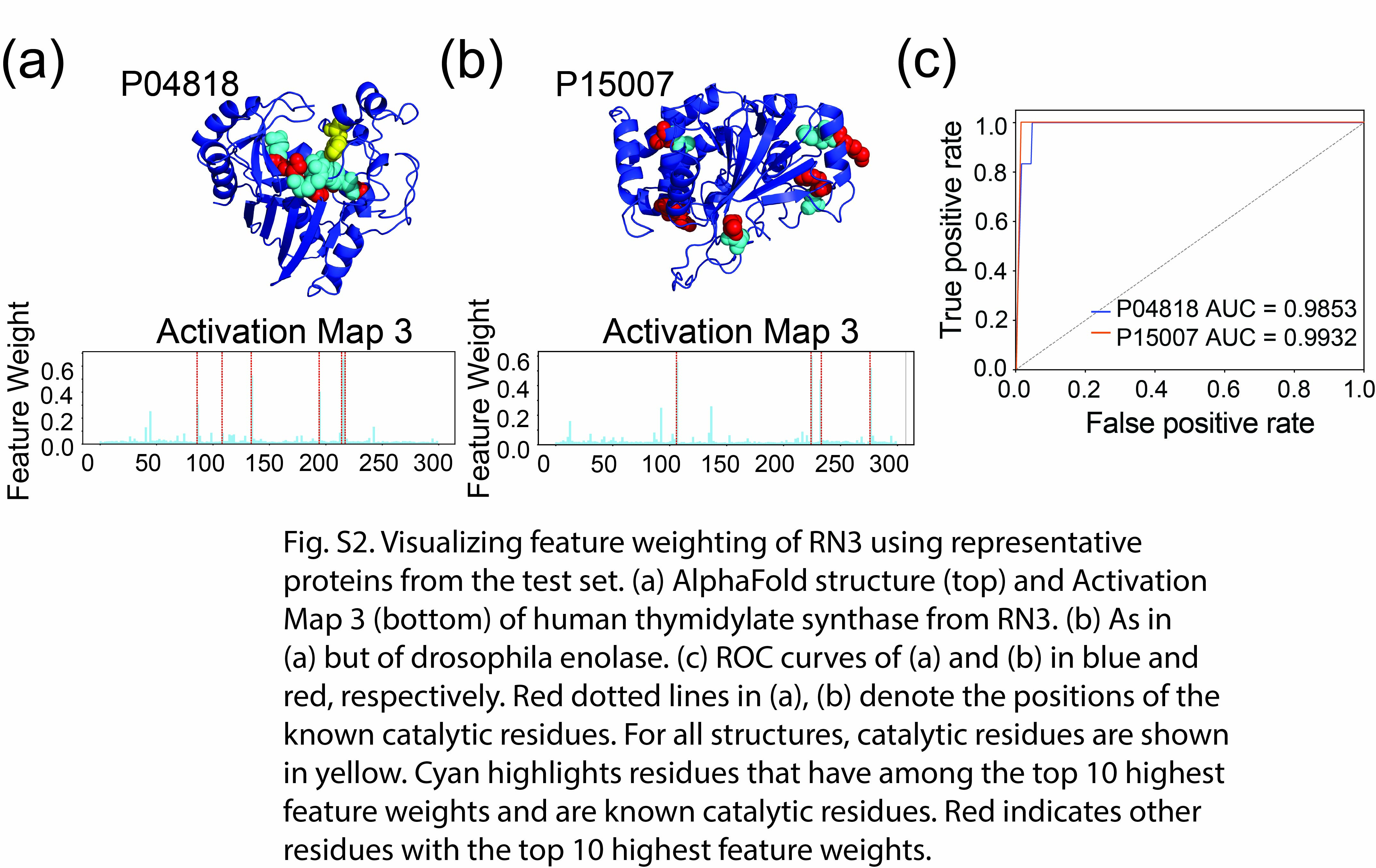

Supplement: btaf190_Supplementary_Data [file btaf190_supplementary_data.zip › btaf190_Supplementary_Data/Kim.50.sup.2.jpg]

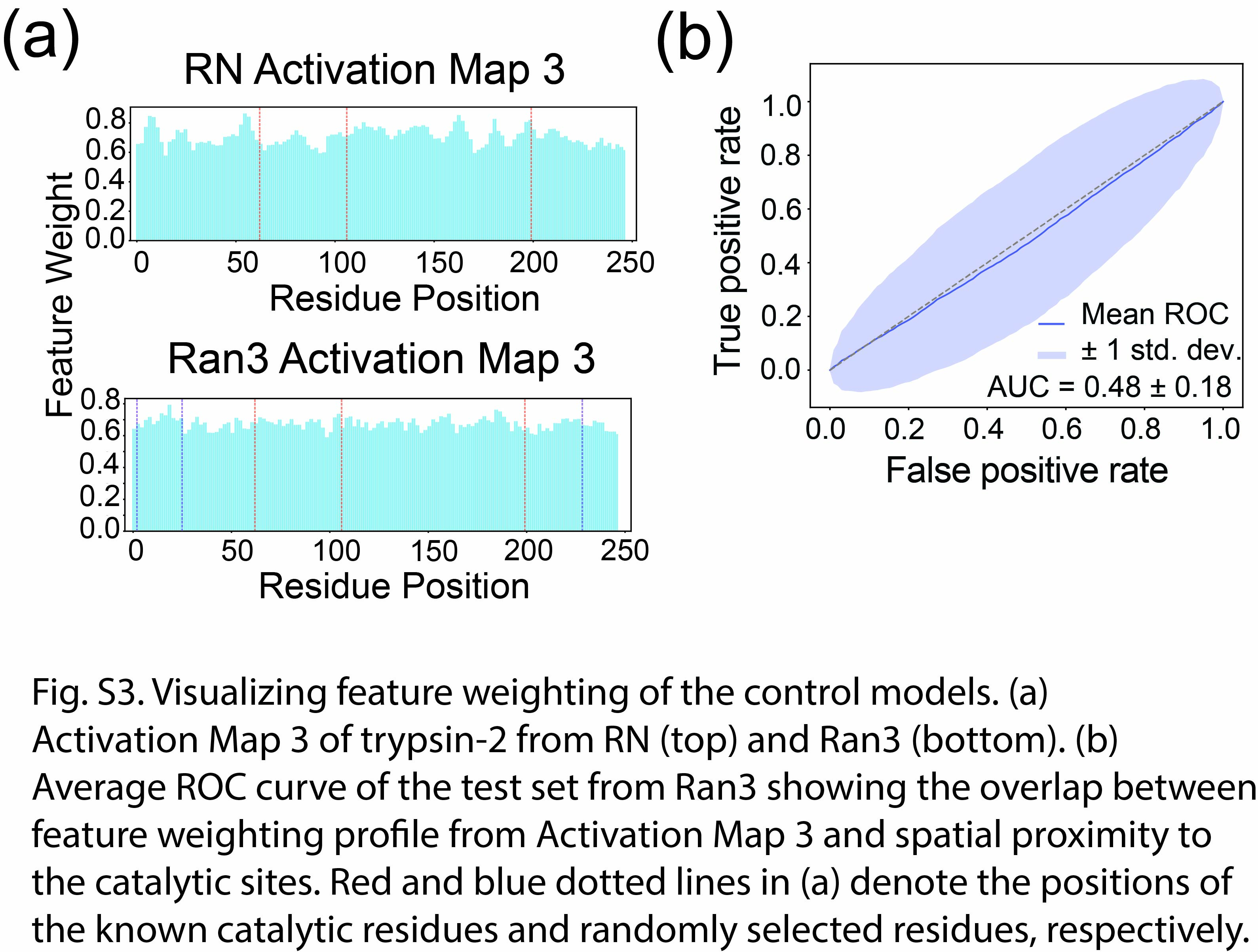

Supplement: btaf190_Supplementary_Data [file btaf190_supplementary_data.zip › btaf190_Supplementary_Data/Kim.50.sup.3.jpg]
